# Effects of Topical Phenytoin Cream on Episiotomy Repair in Primiparous Women: A Double Blind Clinical Trial

**Published:** 2018

**Authors:** Hamideh Pakniat, Atyeh Bahman, Farideh Movahed, Niloofar Mohammadi

**Affiliations:** *Department of Obstetrics and Gynecology, Faculty of Medicine, Qazvin University of Medical Sciences, Qazvin, Iran.*

**Keywords:** Episiotomy, Phenytoin, Betadine, Primiparity

## Abstract

Episiotomy is the most prevalent obstetrical procedure with the purpose of either widening vaginal outlet or helping the fetus to deliver as soon as possible with the most feto-maternal safety. The aim of this study is to find out the effects of topical phenytoin cream on wound repair in primiparous women. One-hundred-thirty primiparous mothers were referred to Kowsar Hospital in Qazvin province participated in this clinical trial. Sixty-five participants were assigned in each of intervention and control groups. The intervention group was treated with topical 1% Phenytoin cream and 10% povidone-iodine (betadine) solution and the control group received placebo and betadine solution. Wound irrigation with betadine was performed as the routine order in the hospital, three times daily and two centimeters of topical phenytoin or placebo cream, were applied to the wound twice daily. The rate of episiotomy repair was measured by REEDA index in the first 24 h, the fifth and the tenth puerperal day. Data analyses were done *t*-test and chi-square test and Mann-Whitney. In the first 24 h, it was 6.43 ± 2.15 in the intervention group versus 6.52 ± 5.09 in the control group with no significant difference. However on The 5^th^ day, it appeared 4.56 ± 3.01 in the intervention group versus 6.54 ± 2.98 in the control group (*p* < 0.001), likewise it was 5.82 ± 2.83 in the control group on the tenth day (*p* < 0.001). Significant difference was detected both in the 5^th^ and 10^th^ postpartum days. The result of this trial suggested that 1% phenytoin cream speeds-up the wound healing process; therefore it could be applied for accelerating episiotomy repair.

## Introduction

 Pregnancy termination with feto-maternal safety is the final goal of obstetrics, because their health is associated with community health. For this purpose, such intervention is made for the sake of mother and her fetus in natural vaginal delivery (1). One of the interventions in natural vaginal delivery is episiotomy, which is a straight incision into the perineal body or posterior vaginal wall to facilitate second stage of labor in almost 15-95% of deliveries (2). If it is done vertically downward, named midline episiotomy and if it starts from midline and continued laterally and far from rectum, is named mediolateral episiotomy (1). Literally lacerations varying from perineal skin to anal sphincter and rectal mucosa can happen in the course of vaginal delivery, especially with precipitous fetal head deceleration, which can be prevented by a prophylactic straight surgical incision with scissors or a surgical knife by the obstetrician in the individualized patients, whereas there is no substitute for surgical judgment and common sense in applying the procedure by the obstetrician (1, 3).

 Due to its physiologic, psychological and socio-economic effects on women, the technique and its afterward nursing are important (4, 5). Although the routine use of episiotomy for vaginal delivery is attenuated currently, its exact prevalence is unknown in Iran. It has been demonstrated that ragged lacerations and tears are more probable in Asian women due to their short, thick perineal tissue (6). Infection, bleeding and dyspareunia are some of the complications of episiotomy. If there were no disruptive factors such as infection or extensive episiotomy. It repairs completely in 3 puerperal weeks (1). Some other complications are restricted perineal motion and scar esthetics, also sexual dysfunction due to delayed wound healing (1, 7). Perineal pain and discomfort due to wound, could postpone emotional infantomaternal contact (8). So, various suggestions such as perineal hygiene, keeping the wound free of moist and some other medical and non-medical recommendations are described to relief the pain and accelerate wound-repair (9). Wound healing process is a complex of new tissue replacing the injured one, including inflammation, coagulation, proliferation, and tissue reorganization. Inflammation is necessary for cleaning the wound by macrophages phagocytizing infectious debris. Their lack results in delayed wound healing (7, 10). Betadine scrub is routinely instructed to the patients in most hospitals currently. It is an aseptic solution, impeding wound infection, therefore accelerate its repair (1). Controversies are present on betadine efficacy. Fata’s study revealed its further side effects comparing with clotrimazole and nistatin in fungal vaginitis (11). Comparing betadine with water also showed no preference of betadine for episiotomy repair in Zahrani’s study (12). Phenytoin is an anticonvulsant non-sedative agent, studied as a topical cream in skin lacerations, diabetic foot, pressure ulcer, and leprosy as well as oral and dental injuries. All of these literatures approved its usefulness (13).

 This agent was first applied to control convulsive attacks on 1937 and gingival hyperplasia has been demonstrated as its known side-effect. This agent could activate fibroblastic proliferation, attenuate collagenase activity, inhibit glucocorticoid production, increase granulation tissue, activate angiogenesis and decrease microbial infection for accelerating wound repair (14-16). It also activates fibroblastic proliferation by accelerating estrogen (17). Phenytoin wound-repair effect was first studied by Shapiro on 1958 in healing gingival surgery sites (18). Some other studies about phenytoin influence on wound-repair acceleration in rodents have been performed which confirm this effects (7). Sehat’s study in 2012, on 120 primiparous, 18-35 years women, demonstrated accelerated episiotomy healing with topical phenytoin cream (17). Lavaf’s study on 2015 was done with the purpose of comparing phenytoin cream and honey effect on episiotomy repair in primiparous participants and concluded that the both are effective, but honey cream effect is more significant (19). In Panahi’s study on 2015, the effect of the combination of olive oil_aloevera was compared with topical phenytoin cream on chronic ulcerous, which revealed that the olive oil-aloe Vera combination has the same biologic effect on chronic-ulcer-repair and also their pain attenuation (20). Considering episiotomy has side-effects such as infection, restriction of motion and scar esthetics; and phenytoin efficacy in accelerating wound repair and also high prevalent of episiotomy in Kowsar medical Educational Hospital, we decided to study topical phenytoin effect on episiotomy repair in primiparous vaginally delivering women.

## Experimental

*Methods*

The study was a prospective, double-blind, and placebo-controlled trial. Two parallel groups of primiparous vaginally delivered mothers with episiotomy were randomly selected from Qazvin Kowsar hospital and this population participated in the study from May 2015 to September, 2015.

Participants and setting eligible participants for the study included primiparous between 18 to 35 years of age, with singleton, term pregnancies from 37 to 42 weeks of gestational age with no underlying disorders such as anemia, cardio-pulmonary, renal or depressive disorders, and with no alcohol or cigarette consumption with normal Body Mass Index.

Subjects were disqualified if any of the followings happened in the course of the study: Hematoma; infection; prolonged labor; episiotomy extension; massive postpartum hemorrhage; postpartum manual placental removal; any perineal, cervical or uterine manipulation or patient’s dissent from continuing cooperation.

The patients have been informed completely, and then the written informed consents were taken. The trial was approved in the ethics committee of Qazvin medical university. The study was registered in the Iranian registry of clinical trials (registration No. IRCT 2015 10118611N1). In order to preserve the double-blind condition, phenytoin cream and placebo were dispensed in identical-appearing 30 g coded tubes. To provide the placebo, a contract to make 70 identical tubes of placebo, just like the tubes for phenytoin cream, was made with Daru-Pakhsh Company.

*Procedures*

**Figure 1:**
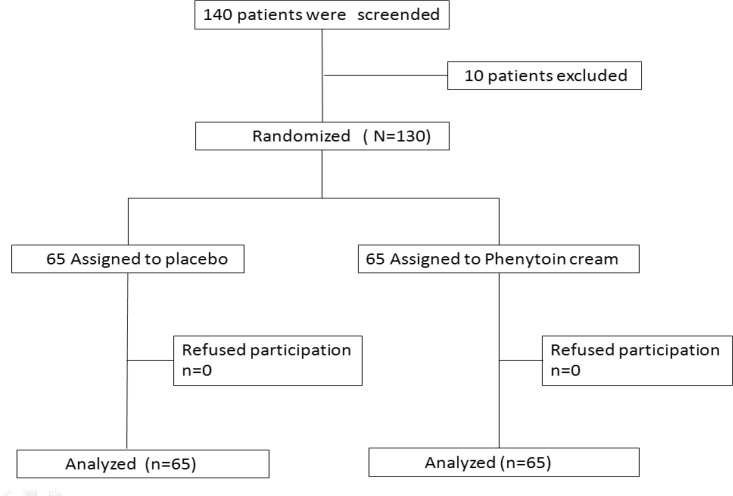
Consort flow diagram

After written informed consents were gathered from the patients, participants entered in one of the parallel groups through randomized, double blind, clinical trial. Through using computer-generated list of random numbers, the patients were randomly assigned in two groups. Then the questionnaires of personal-obstetrical data were filled in the labor room. At the end of episiotomy suturing, the obstetrical-ward researcher, instructed the necessary tips about wound care and emphasized on personal hygiene and adequate nutrition. The randomly assigned participant received 1% phenytoin cream or placebo cream in coded 30 g tubes, with 10% betadine solution for both (either the intervention group or the control group). One-hundred-thirty primiparous participants were enrolled in this trial. They were randomly assigned in one of the two groups, 65 in each (as it was calculated in the statistical evaluation). They would rather first clean the perineum, next dry the area before applying 2 centimeters of the cream (almost at the size of fingertip) on the wound twice daily. Also they should scrub with betadine solution three times a day, as routine in the hospital. The first wound repair evaluation was performed in the next 24 h of delivery and filled in the questionnaire. The mothers were encouraged for the 5^th^ and 10^th^ day visits in the hospital. They were all evaluated by one of the researchers and the wounds healing process were filled in the questionnaire on the basis of REEDA index. REEDA index is an international index for evaluating episiotomy wound repair. It is the abbreviation of five criteria, including: redness, edema, ecchymosis, discharge, and approximation of wound edges. Each of these has 0-3 points, and the summation is 0-15. A lower index represents a better wound repair. A higher index is the representative of poor wound healing (Figure 1).

*Statistical analysis*

Data were collected and analyzed using SPSS version 19, by student’s *t*-test and Mann Whitney test. Statistical significance was defined at *p*-value < 0.05. Repeated measures analysis of REEDA indices means was used to assess the effect of treatment.

## Results and Discussion

One-hundred-thirty primiparous women enrolled in this study, Mean maternal age was 23.9 ± 4.86. Mean gestational age at termination was 38.72 ± 5.62 weeks. Mean newborn weight was 3210 ± 49 g. All of participants assigned to two groups of sixty-five members. The demographic information for each group separately have been summarized and shown in Table 1.

**Table 1:** Information of primigravidas in both phenytoin and control groups

**Variable**	**phenytoin** ** (** **n ** **= 65** **)** **Mean ± SD**	**control** ** (** **n = 65** **)** **Mean ± SD**	***P*** **-value**
Age (years)	5.26 ± 24.63	4.35 ± 23.18	0.090
Gestational age (weeks)	4.77 ± 38.54	6.35 ± 38.88	0.360
Neonate weight (g)	493.1 ± 3294.6	477.7 ± 3130.8	0.061
Systolic blood pressure (mmHg)	8.02 ± 110.71	7.97 ± 10824.92	0.184
Diastolic blood pressure (mmHg)	7.58 ± 71.90	7.30 ± 68.69	0.19
(mg/dL) Hemoglobin	0.75 ± 11.55	1.30 ± 11.79	0.208
Temperature (°C)	0.15 ± 36.97	0.21 ± 37.0	0.598
Heart rate (per min)	4.53 ± 83.4	4.93 ± 84.51	0.068
BMI	4.98 ± 28.65	3.90 ± 28.55	0.904

 Systolic and diastolic blood pressure, body temperature, heart rate, serum hemoglobin and Body Mass Index in the two groups were evaluated and compared. The two groups were well matched and there were no statistically significant differences between the groups in demographic or any of the mentioned variables above. There were also no significant difference between the groups in the duration of the 1^st^ and second stages of labor, membrane rupture, episiotomy suturing; or vaginal exam frequency, the number of stiches, desired or undesired conception and maternal carrier. If the mother rejected continuing her cooperation in the study, a replacement of an eligible participant would be done. We observed no significant difference in REEDA Indices of the first 24 h of delivery. They were 6.43 ± 2.15 in phenytoin group and 6.52 ± 5.09 in placebo group (Table 2).

**Table 2:** Compared mean of REEDA scores on the first, fifth, tenth days after delivery

**Variable**		**case** ** (** **n = 65** **)** **Mean ± SD**	**control** ** (** **n = 65** **)** **Mean ± SD**	***P*** **-value**
REEDA Index	first 24 h	2.15 ± 6.43	5.09 ± 6.52	0.46
	5^th^	3.01 ± 4.56	2.98 ± 6.54	<0.001
	10^th^	2.28 ± 2.50	2.83 ± 5.82	<0.001

**Figure 2:**
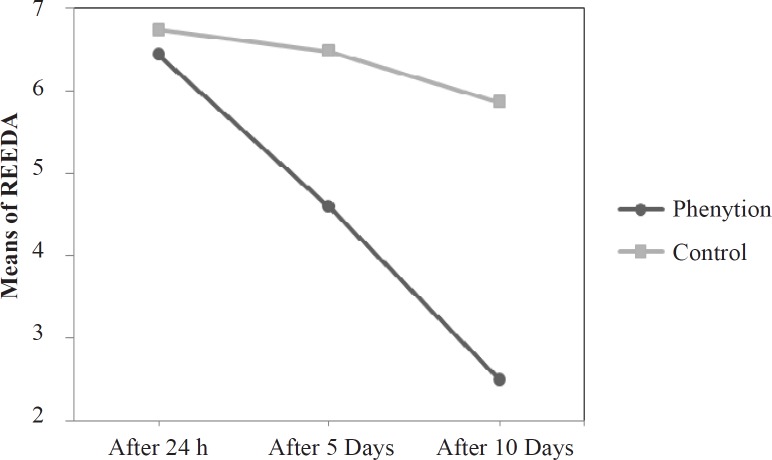
Compared means of REEDA scores on the first, fifth, tenth days after delivery

 Except for the parameter of approximation, this demonstrated significant difference in the first 24 h. In spite, Five-parameter REEDA Index comparison, revealed significant difference on the fifth and tenth day. The indices were 4.56 ± 3.01 in phenytoin group and 6.54 ± 2.98 in placebo group on the 5^th^ day; they were 2.50 ± 2.28 in phenytoin group and 5.82 ± 2.83 in placebo group on the 10^th^ day (Figure 2).

To compare REEDA indices of the three time-durations, defined for the wound evaluation, repeated measures analysis of the means was used (Table 3).

**Table 3:** Compared mean of REEDA scores between the two groups in three different periods of separation criteria

**Variable**		**Phenytoin** **(** **n = 65** **)** **Mean ± SD**	**Control** **(** **n = 65** **)** **Mean ± SD**	**Z**	***P*** **-value**
Redness	first 24 h	0.59 ± 1.27	1.01 ± 1.43	0.475	0.633
	5^th^	0.67 ± 1.01	0.72 ± 1.29	2.23	0.026
	10^th^	0.78± 0.72	0.76 ± 1.27	3.978	<0.001
Edema	first 24 h	0.66± 1.27	1.15 ± 1.46	0.856	0.392
	5^th^	0.83± 0.92	0.76 ± 1.30	2.946	0.003
	10^th^	0.70 ± 0.46	0.70 ± 1.24	5.768	<0.001
Ecchymosis	first 24 h	0.73 ± 1.33	1.36 ± 1.50	0.665	0.506
	5^th^	0.80 ± 0.78	0.88 ± 1.15	2.457	0.014
	10^th^	0.56 ± 0.49	0.08 ± 1.12	4.700	<0.001
Discharge	first 24 h	0.78 ± 1.28	1.30 ± 1.42	0.491	0.623
	5^th^	0.88 ± 0.80	0.83 ± 1.26	3.244	0.001
	10^th^	0.60 ± 0.38	0.63 ± 0.96	5.320	<0.001
Approximation of wound edges	first 24 h	0.65 ± 1.30	1.13 ± 2.04	4.364	<0.001
	5^th^	0.90 ± 1.04	0.81 ± 1.46	2.779	0.005
	10^th^	0.63 ± 0.44	0.84 ± 1.24	5.596	<0.001

We observed no side effects during the study period in any of the subjects and all of them had good compliance to the treatment. Some transient epidermal irritations have been reported in some studies, which required no intervention. Generalized rashes and itching have disappeared by the drug discontinuation in these studies (19).

Results of this study revealed accelerated wound repair in phenytoin group, in comparison with placebo. In our study, demographic data including maternal age, gestational age at delivery, newborn weight, systolic and diastolic blood pressure, serum hemoglobin, body temperature, heart rate, Body mass index, duration of the first and second stages of labor, membrane rupture, episiotomy suturing, or vaginal exam frequency, and the number of stiches disclosed no significant difference between the two groups which correspond with Sehati’s, Golezar’s, Golmakani’s and Lavaf’s studies (5, 9, 17 and 19).

REEDA Indices compartment between the two groups in the first 24^h^ of delivery demonstrated no significant difference, though it was less in the intervention group (6.43 ± 2.15 *vs*. 6.52 ± 5.09). Sehati’s study disclosed less REEDA Index in the phenytoin group versus control group in the first 24 h (4.81 ± 1.87 *vs.* 5.07 ± 1.89) (17). Lavaf’s study showed no significant difference of REEDA indices between the groups on the first puerperal 24 h which was in correspondence with ours (19). This study also proved significant difference of mean REEDA Indices between the two groups on the 5^th^ and 10^th^ puerperal days (on the 5^th^ day, 4.56 ± 3.01 for phenytoin versus 6.54 ± 2.98 for placebo and on the 10^th^ day, 2.50 ± 2.28 for phenytoin versus 5.82 ± 2.83 for placebo). Sehati’s study disclosed significant difference of mean REEDA indices between the two groups on the 10^th^ puerperal day. Although approximation of wound edges was less in Sehati’s study, it had no significant difference. (Intervention: 1.15 ± 1.1 *vs.* control 4.01 ± 1.43) (17). Lavaf’s study also approved that phenytoin plus honey cream was more efficient than placebo on the 7^th^ puerperal day; however, honey cream was more efficient than phenytoin cream. (phenytoin 91/5%, honey 93/9%; *vs.* placebo 78/9%) (19). The both trials were in accordance with ours.

An experiment on tissue texture during wound healing made it known that phenytoin cream application is associated with reduction in plasma cells and inflammatory cells, though fibroblasts, angiogenesis, and collagen synthesis increase. Collagenase suppression by phenytoin is not a direct inhibitory achievement, but is achieved by decreasing glucocorticoids synthesis and competitive antagonism (7). It also regulates connective tissue metabolism, accelerating fibroblasts proliferation, increasing Platelet Activating Factor (PAF) and decreasing polymorphonuclear and eosinophil infiltration (7, 21). Another similar study has been performed applying turmeric ointment (curcuma) with chamomile essence and Brome lain tablets for accelerating episiotomy repair (5, 6 and 9). Golmakani’s study revealed turmeric ointment could decrease REEDA Indices in comparison with placebo on the 7^th^, 10^th^, and 14^th^ puerperal days (5). Golezar’s study by comparing REEDA indices showed that consuming bromelain tablets would accelerate episiotomy repair in comparison with placebo on the 3^rd^, 7^th^ and 10^th^ puerperal days (9). Pazandeh *et al. *compared chamomile essence and placebo to evaluate their wound healing efficacy but they found no significant difference (6). Carneiro, on 2003, carried out a research with the purpose of comparing phenytoin cream and eusol in non-malignant chronic foot ulcer which disclosed accelerated granulation tissue formation and wound healing with phenytoin (22). Elnahas *et al.* on 2009, studied topical phenytoin cream effect on diabetic foot, which approved its efficacy (23). It also attenuated wound bacterial load and its application would have antibacterial effects for staphylococcus aureus, E. coli and Kellebsiella after 7-9 days of utilization (7). Emad Hokkam, on 2012, studied phenytoin cream effect on chronic venous ulcers, which approved its efficacy (24).

 Topical phenytoin cream, not only accelerates wound repair and anti-inflammatory reactions, but also adjusts pH and increases wound blood supply (17). Rashidi on 2012 conducted a comparative study of phenytoin and betadine about wound pain attenuation on 120 primiparous women in Tabriz Medical Center. VAS criteria in the 1^st^ 24 h and 10^th^ puerperal day, demonstrated significant phenytoin cream role on pain attenuation in comparison with betadine (25).

There are various animal studies on the subject of phenytoin cream role for epidermal wound healing. Riahi on 2009 conducted a research with the purpose of comparing 1% phenytoin cream and unrefined honey with Vaseline, in accelerating an open wound repair at inflammatory and reorganization phases in rats. The study proved not only more facilitated wound repair with honey and phenytoin creams in comparison with Vaseline, but also more accelerated wound repair with phenytoin cream comparing with honey cream (7). Sengupta on 2015 managed a study of comparing 1% and 2% phenytoin powder on epidermal wound in rats which revealed acceptable epithelialization in both, but more accelerated repair with 2% phenytoin powder (26). Omidian’s research on 2015, demonstrated that phenytoin cream and 5% quince seed had similar efficacy in tissue repair (27). However, there exist some studies opposing phenytoin cream effect on wound repair process (28, 29).

Our limitations were related to personal hygienic state and wound care variation which controlled researcher’s close following-up and repeated instructions.

Our study showed that phenytoin application causes earlier episiotomy wound healing which was in accordance with the former studies. One of the advantages of this study was the evaluation of the wound on the 5^th^ postpartum day. Comparing the two groups proved that as the wound heals earlier, the maternal satisfaction is more and this is an important point in conducting governmental goals and guidelines to persuade the mothers and also increasing natural vaginal delivery, therefore decreas cesarean/hysterotomy rate and its complications and economic load.

Another advantage of this study was reinforcing the health centers to refer to their under-cover population for obstetrical visit on the 5^th^ and 10^th^ postpartum days. All of these patients were visited and examined by one of the researchers of the study in the hospital private clinic.

Considering the inclusion and exclusion criteria, our sample was almost homogenous.

Our limitations were lack of personal hygiene and appropriate nutrition in some of the participants. On the other hand, some of the mothers rejected continuing their cooperation suddenly and with no rational reason and we had to replace them and perform all steps again.

## Conclusion

The results of this study disclosed phenytoin cream efficacy on accelerating episiotomy wound repair. Considering natural vaginal delivery increment and consequently further episiotomies, we recommend sodium-phenytoin cream scrub over Bethadine due to its lower cost, more availability, ease of use, and comfort ability with facilitating wound repair without serious side-effects or

complications.
